# Identification of Biomarkers Affecting Cryopreservation Recovery Ratio in Ram Spermatozoa Using Tandem Mass Tags (TMT)-Based Quantitative Proteomics Approach

**DOI:** 10.3390/ani13142368

**Published:** 2023-07-20

**Authors:** Chunhuan Ren, Zhipeng Sun, Yale Chen, Jiahong Chen, Shijia Wang, Qingqing Liu, Penghui Wang, Xiao Cheng, Zijun Zhang, Qiangjun Wang

**Affiliations:** 1 College of Animal Science and Technology, Anhui Agriculture University, Hefei 230036, China; renchunhuan@ahau.edu.cn (C.R.); fsunzhipeng@163.com (Z.S.); chenyale1@163.com (Y.C.); wangshijia1014@163.com (S.W.); qingqingliu04@163.com (Q.L.); wangpenghui15987@163.com (P.W.); chengxiao2533@163.com (X.C.); 2 New Rural Develop Research Institute, Anhui Agricultural University, Hefei 230036, China; chenjiahong@ahau.edu.cn; 3 Center of Agriculture Technology Cooperation and Promotion of Dingyuan County, Dingyuan 233200, China

**Keywords:** spermatozoa, cryopreservation, tandem mass tags (TMT), proteomics, bioinformatics, ram

## Abstract

**Simple Summary:**

Sperm cryopreservation is one of the key procedures in artificial insemination technology. With the continuous development of cryopreservation technology, the survival rate of frozen sperm has been greatly improved, and it is widely used in artificial insemination technology. However, the molecular regulation of sperm cryopreservation recovery ratio in rams remains poorly understood. The main objective of this study was to gain a better understanding of the biomarkers that play a role in sperm freezing tolerance in the proteome levels of the rams. Our results confirm that some proteins and pathways associated with high and low cryopreservation recovery ratios were identified. These findings related to sperm freezing tolerance improve our understanding of molecular mechanisms of sperm resistance to low-temperature environments, and are helpful for livestock breeding.

**Abstract:**

Sperm proteins play vital roles in improving sperm freezing resilience in domestic animals. However, it remains poorly defined which proteins regulate the freezing resilience of spermatozoa in rams (*Ovis aries*). Here, we compared the proteome of ram sperm with a high cryopreservation recovery ratio (HCR) with that of ram sperm with a low cryopreservation recovery ratio (LCR) using a tandem mass tag-based quantitative proteomics approach. Bioinformatic analysis was performed to evaluate differentially expressed proteins (DEPs). A total of 2464 proteins were identified, and 184 DEPs were screened. Seventy-two proteins were higher in the LCR group. One hundred and twelve proteins were more abundant in the HCR group, and they were mainly involved in the regulation of oxidative phosphorylation and thermogenesis pathways. Proteins in high abundance in the HCR group included the S100A family, such as S100A8, S100A9, S100A14, and S100A16, effectively controlling for CA^2+^ and maintaining flagella structure; HYOU1 and PRDX1, which participate in antioxidant protection and anti-apoptosis to prevent cell death; and HSP90B1, which maintains cell activity and immune response. Our results could help illuminate the molecular mechanisms underlying cryopreservation of ram semen and expand the potential direction of cryopreservation of high-quality semen.

## 1. Introduction

The cryopreservation process of sperm has an irreversible impact on sperm vitality, acrosome reaction rate, morphology, and egg fertilization rate [1]. However, some ram semen exhibits tolerance to cryopreservation stress. Semen cryopreservation is used in assisted reproductive techniques and motile spermatozoa are always selected to be injected into oocytes, ensuring viability and increasing the probability of fertilization [2,3]. The cryopreservation recovery ratio of sperm is an important index to judge the effect of cryopreservation on sperm and an important factor to determine the success of artificial insemination [4]. The cryopreservation recovery ratio of sperm varies greatly with individuals, and few semen samples always present poor cryopreservation resistance before freezing. However, predicting the freezing ability of spermatozoa using conventional sperm quality parameters prior to freezing is not rigorous [5]. Mature spermatozoa are transcriptionally inactive [6]. Therefore, it is necessary to identify potential biomarkers that can accurately predict sperm freezing resilience, and an accurate selection of semen samples based on these biomarkers is an excellent guide for improving semen cryopreservation.

Previous work has started predicting sperm freezing resistance markers through the study of sperm proteomics. Thurston et al. found genomic markers of sperm freezing resistance by the evaluation of amplified fragment length polymorphism (AELP) [7]. Yoon and Kwon et al. identified potential biomarkers affecting sperm freeze tolerance in bulls and wild boar, respectively [8,9]. Villagran et al. reported that voltage-dependent anion-selective channel protein 2 (VDAC2) can be a marker to predict the freezing tolerance of pig sperm before freezing, and that both acrosin binding protein and triosephosphate isomerase are related to sperm motility [10,11]. In rams, the decrease of protein expression levels of hexokinase1 (HXK1) and casein kinase II subunit alpha (CSNK2A2) in sperm flagellum may affect sperm motility following freeze–thawing [12]. Numerous proteins in sperm and seminal plasma may affect how resistant human sperm is to cryopreservation, according to proteomics investigations of human semen [13]. However, there have been few studies linking the recovery of frozen ram sperm to the protein composition of the sperm. While the diversity of proteins in ram sperm is well known, the underlying biomarkers of the sperm cryotolerance are still unknown.

In our previous work, the control factors behind the physiological and biochemical mechanisms of sperm viability were identified based on the analysis of proteomic data [14]. High throughput proteomics (tandem mass tags (TMT) combined with liquid chromatography–mass spectrometry (LC-MS/MS)) was used to analyze the proteome of sperm in our study, aiming to reveal the seminal proteomic profile of Dorper Sheep rams (*Ovis aries*) and identify the protein markers associated with sperm cryopreservation recovery ratio. These proteins, related to sperm freezing tolerance, improve our understanding of the regulatory mechanism of sperm resistance to low-temperature environments, and these differentially expressed proteins may be important for sperm cryopreservation.

## 2. Materials and Methods

### 2.1. Experimental Design

**Figure 1:**
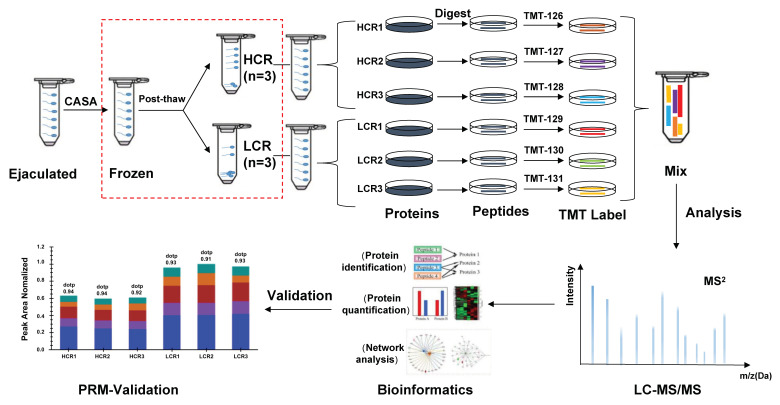
The workflow showing use of TMT technology to identify the markers affecting sperm cryo-resuscitation ratio.

Specimens were prepared using fresh ejaculates from six rams. Briefly, fresh ejaculates were collected in four replicates from 6 rams using an artificial vagina, and the motility of each ejaculate was assessed via a computer-assisted sperm analyzer (CASA, sperm class analyzer-5.4.0.0; MICROPTIC Supply, Barcelona, Spain) to ensure pre-freezing motility exceeding 80%. Then, the cryopreservation recovery ratios for the 6 rams were ranked; 3 rams were assigned into high cryopreservation recovery ratio (HCR) and the other 3 rams were divided into low cryopreservation recovery ratio (LCR) groups based on their survival ratio. Sperm proteomes were investigated using TMT-based protein labeling, LC-MS/MS identification, and relative protein quantification. Finally, quantification results were confirmed by parallel reaction monitoring (PRM). Differentially, expressed proteins (DEPs) were identified and functionally classified. The workflow for the global profiling experiments is displayed in Figure 1.

### 2.2. Animals and Ethics

Mature Dorper rams (*Ovis aries*), approximately 3 years old and similar in weight (Table 1, Supplementary Table S1), were used for semen collection and were housed at the Jianghuai Watershed Comprehensive Test Station, Anhui Agricultural University (Anhui, P.R. China). Animals were maintained on an artificial mixed grassland (comprising *Dactylis glomerate* L., *Medicago sativa* L., *Cichorium intybus* L., *Lolium perenne* L., *Trifolium repens* L.) grazing diet for 5 months, supplemented with eggs and carrots to improve sperm motility, and with free access to water. All experimental protocols according to established standards were approved by the Animal Care Committee, Anhui Agriculture University (AHAU2022009).

### 2.3. Sample Collection and Preparation

Semen was obtained from each ram using an artificial vagina following standard procedure [15]. Firstly, we collected ejaculates (*n* = 4/ram) from 12 healthy adult rams with similar ages and weights. Computer-aided sperm analysis (CASA) was used to assess the total and progressive motility under an imaging system (Basler acA780-75gc, Ellen Dalph, Germany) based on chamber slides (Goldcyto, SCA 20-04-01, Barcelona, Spain) and light microscopy (Nikon Eclipse-E200, Tokyo, Japan) at a 100× magnification, according to the World Health Organization (W.H.O) criteria. The program setting was established as follows: frames acquired, 25; frame rate, 30 Hz; coherence, 12; minimum contrast 80, minimum velocity of average path 30 μm/s. The fresh ejaculates were diluted with phosphate-buffered saline (pre-warmed) according to the ratio of 1:10 before CASA analysis, and a hemocytometer was used to measure the concentration. Only fresh ejaculates with motility > 80%, deformity rate < 15% [16], and sperm concentration over 3 × 10^9^/mL were accepted. Then, each ejaculate was collected and divided into two fractions (across 4 replicates). One fraction was used as a control to prepare a spermatozoa sample through centrifugation (1600× *g*, 30 min, 4 °C) to remove seminal plasma, and was then washed three times with ice-cold phosphate-buffered saline (PBS) to remove other contaminates, quick-frozen in liquid nitrogen and stored at −80 °C for protein extraction. The other fraction was frozen and resuscitated, respectively. Finally, spermatozoa samples were collected from high (HCR, *n* = 3 rams; 4 ejaculates/ram) and low (LCR, *n* = 3 rams; 4 ejaculates/ram) cryopreservation recovery ratio groups, respectively.

Identification of HCR and LCR rams was made as follows: pre-warmed extender containing 75% Optidy 1 (Cryo-vet, L’Aigle, France) and 25% ultra-pure water was slowly added to semen at a ratio of 8:1 (diluent: semen, *v*/*v*). Diluted samples were chilled to 5 °C from 37 °C in the refrigerator over 3 h; then, the pre-freezing motility was assessed (only those samples with pre-freeze motility >80% were accepted) and the straw method was used to freeze the semen. Equilibrated samples were loaded into pre-cooled 0.25 mL straws (IMV, Legrad, France) and sealed with polyvinyl chloride (PVC, Arkema, Paris, France) powder. Straws were placed on a pre-chilled freezing rack 3 cm above the liquid nitrogen surface for 8 min. Following freezing, all straws were plunged into liquid nitrogen for storage until assessment. Each straw was thawed for 1 min in a 37 °C water bath with agitation, and then the motility and motion kinematics parameters (WHO and Kruger Standards) of cryopreserved spermatozoa were evaluated using the CASA system [17]. Spermatozoa were assessed for percent sperm motility (MOT), curvilinear velocity (VCL), straight line velocity (VSL), average path velocity (VAP), linearity (LIN), straightness (STR), lateral head displacement (ALH) and beat cross frequency (BCF), using the CASA software (sperm class analyzer-5.4.0.0; MICROPTIC Supply, Barcelona, Spain). The percent motility before (no significant difference in sperm motility, *p* > 0.05) and after freezing (significant difference in sperm motility, *p* < 0.05) was compared to calculate the cryopreservation recovery ratio. Cryopreservation recovery ratios for 12 rams were then ranked, and the 3 rams which consistently recorded more than 60% of cryopreservation recovery ratio were classified into the HCR group, and the other 3 rams which consistently recorded less than 30% of cryopreservation recovery ratio were classified into the LCR group. Meanwhile, other rams’ spermatozoa samples that did not fit these conditions were discarded. To increase sperm volume and eliminate variability between samples, four replicates of each ram were performed and the means were calculated, and all experiments were performed with three rams of the HCR and LCR group, respectively. Finally, four ejaculates from each ram were pooled together.

### 2.4. Quantitative Mass Spectrometry Analysis

#### 2.4.1. Protein Extraction and Digestion (PTM)

All spermatozoa samples were first sonicated on ice three times in lysis buffer (8 M urea, 1% Protease Inhibitor Cocktail) with a high intensity ultrasonic processor (Scientz). For PTM experiments, inhibitors were also added to the lysis buffer; 3 μM TSA and 50 mM NAM were used for acetylation. Samples were centrifuged at 12,000× *g* for 10 min at 4 °C to remove the remaining fragments. Then, the supernatant was collected, and the concentration of protein was determined using a BCA Kit (Bio-Rad, Hercules, CA, USA), according to the manufacturer’s protocol. Finally, each electrophoresis channel was loaded with 20 µg of protein through 10% (*w*/*v*) SDS-PAGE detection.

Protein digestion was performed as follows: an equal quantity of protein solution was reduced and incorporated into 5 mM dithiothreitol at 56 °C for 30 min, and RT was alkylated with 11 mM iodoacetamide for 15 min in a dark environment. Protein sample was diluted to a urea concentration of less than 2 M by adding 100 mM tetraethylammonium bromide (TEAB). At last, the protein suspensions were digested with a mass ratio of 1:50 trypsin to protein overnight at 4 °C, and the second digestion was performed with a mass ratio of 1:100 trypsin to protein for 4 h.

#### 2.4.2. TMT Labeling

After trypsin digestion, the peptides were desalted using a Strata X C18 SPE column (Phenomenex) and dried by vacuum centrifugation. The desalted peptides (100 µg) from each sample were dissolved in 0.5 moL/L TEAB solution and labeled using the TMT Labeling Kit (ThermoFisher Scientific, Waltham, MA, USA) according to the manufacturer’s protocol. Briefly, a unit of TMT reagent was thawed and reconstituted in acetonitrile. After the polypeptide mixture was incubated at RT for 2 h, it was pooled, desalted, and dried by vacuum centrifugation.

#### 2.4.3. High-Performance Liquid Chromatography (HPLC) Fractionation and LC-MS/MS Analysis

Using a Thermo Betasil C18 column (5 μm particles, 10 mm ID, 250 mm length), peptides were fractionated via high pH reverse-phase HPLC. Briefly, 8% to 32% acetonitrile was used to gradient separate the peptides over 60 min and divide them into 60 fractions. Then, we combined the peptides into six fractions and dried them by vacuum centrifuge. The peptides were combined in 0.1% formic acid (solvent A) and directly loaded onto a reversed-phase C18 analytical column (Thermo Scientific, 15 cm length, 75 μm i.d., Waltham, MA, USA). The gradient included solvent B (0.1% formic acid, 98% acetonitrile) from 6% to 80% for 40 min, and the flow rate of EASY-nLC 1000 UPLC system was 400 nL/min.

Proteomics based on LC-MS/MS was performed by PTM BioLab Co., Ltd. (Hangzhou, China) as described previously [17]. In a Q Exactive TM Plus connected online to the UPLC, the peptides were submitted to an NSI source followed by tandem mass spectrometry (MS/MS). With an NCE setting of 28, peptides were chosen for MS/MS analysis, and fragments were found in the Orbitrap at a resolution of 17,500.

#### 2.4.4. Database Search and Bioinformatic Analysis

The MaxQuant search engine (v. 1.5.2.8) was used to process the MS/MS data. The human UniProt database and reverse decoy database were searched simultaneously using mass spectral data. As a cleavage enzyme that permits up to four missed cleavages, trypsin/P was described. The mass tolerance for fragment ions was set to 0.02 Da, whereas the mass tolerance for precursor ions was 20 ppm in the initial search and 5 ppm in the main search. The alterations of carbamidomethyl on Cys and acetylation and oxidation on Met were designated as constant and changeable, respectively. The minimum score for changed peptides was set at >40 and the FDR was adjusted to 1%.

The UniProt-GOA database, located at http://www.ebi.ac.uk/GOA (accessed on 5 February 2023), served as the source for the Gene Ontology (GO) terms. The InterProScan software (v.5.14-53.0) would annotate a protein’s GO function based on a protein sequence alignment approach if certain discovered proteins were not annotated by the UniProt-GOA database. The protein families, domains, and functional sites were analyzed by InterPro (http://www.ebi.ac.uk/interpro/, accessed on 5 February 2023). In addition, Wolf PSORT was used to predict the subcellular localization of proteins, which is an updated version of PSORT/PSORT II software (v.0.2), used for the prediction of eukaryotic sequences. The Kyoto Encyclopedia of Genes and Genomes (KEGG) database (http://www.genome.jp/kegg, accessed on 10 February 2023) combined with DAVID tool (https://david.ncifcrf.gov/, accessed on 10 February 2023) were used to perform enrichment pathways, and protein–protein interaction analysis was performed for all DEPs based on the STRING database (http://string-db.org, accessed on 10 February 2023).

### 2.5. PRM Validation

For validation of the TMT LC-MS/MS results, 14 proteins were selected among the DEPs. PRM-MS analysis was conducted by the PTM BioLab Co. Ltd. According to the TMT analysis, the 8 μg protein sample was prepared, reduced, alkylated, and trypsin digested. The resulting peptide mixture was dissolved in solvent A, then separated into 7% to 25% solvent B (*v*/*v*) for 40 min, 25% to 35% solvent B (*v*/*v*) for 12 min, 30–80% (*v*/*v*) solvent B for 4 min, and 80% solvent B (*v*/*v*) for the last 3 min. On the Thermo Fisher EASY-nLC 1000 UPLC system, a constant flow rate of 700 nL/min was employed. The peptides underwent tandem mass spectrometry (MS/MS) in a Q ExactiveTM Plus (Thermo) linked online to the UPLC after being subjected to NSI source analysis. In Orbitrap, the complete peptides were found at a resolution of 35,000. The fragments were subsequently identified in the Orbitrap at a resolution of 17,500 after the peptides were chosen for MS/MS analysis with the NCE setting of 27. Peptide settings: enzyme was set as trypsin (KR/P), max missed cleavage was set as two, length was set to 8–25, variable modification was set as Carbamidomethyl on Cys, oxidation on Met, and the max variable modifications were set as three.

### 2.6. Statistical Analysis

The data are presented as mean ± SEM. Statistical analysis of cryopreserved and fresh sperm CASA parameters were performed using a one-way Student’s *t*-test with a significance level *p* < 0.05 using SPSS software (v. 25.0, Chicago, IL, USA). *p*-value < 0.05 was considered statistically significant. GraphPad Prism (V.9.3.1) software (GraphPad Software, San Diego, CA, USA) was used for statistical analyses.

## 3. Results

### 3.1. Comparison of Sample Parameters between HCR and LCR

**Figure 2:**
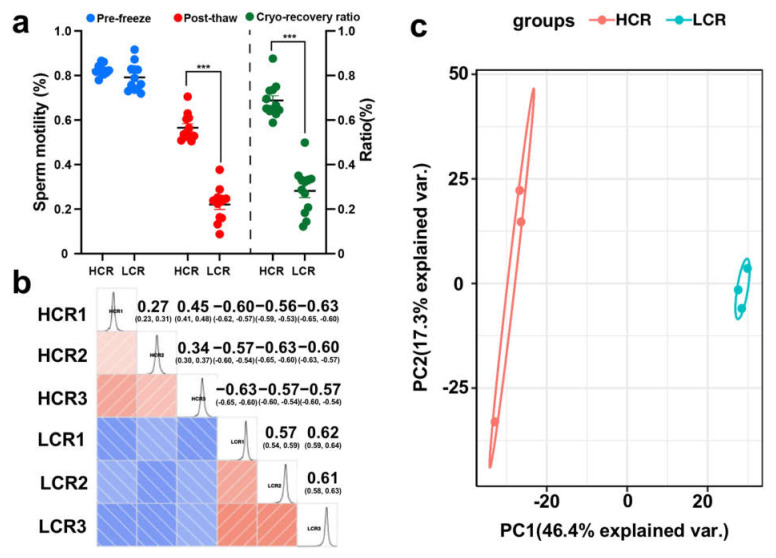
Proteomics analysis of HCR and LCR ratio spermatozoa. (**a**) HCR and LCR sperm groups are represented by different colors. The left *y*–axis indicates sperm motility (%), and the right *y*–axis indicates motility rate (%) of post−thaw and pre−freeze sperm. *** *p* < 0.001. (**b**) Pearson heat map of correlation coefficient between all samples. A Pearson coefficient closer to −1 is a negative correlation, closer to 1 is a positive correlation, and closer to 0 is insignificant (*p* < 0.05). (**c**) Principal component analysis (PCA) of differentially expressed sperm proteins of the two groups; HCR1−HCR3, samples of HCR spermatozoa group; LCR1−LCR3, samples of LCR spermatozoa high group.

The motility and kinematic parameters of spermatozoa in the HCR and LCR groups were analyzed using the CASA system. There were no significant differences between HCR and LCR for motility parameters from fresh samples (*p* > 0.05). Furthermore, kinematics parameters such as MOT, VCL, VSL, LIN, STR, and ALH also showed no significance between HCR and LCR (*p* > 0.05). However, the MOT, VCL, VSL, and ALH showed significant differences (*p* < 0.01) and LIN and STR showed significant differences (*p* < 0.05) between the two groups (Table 2, Supplementary Table S2). In addition, the results showed that the sperm cryopreservation recovery ratio in the HCR group was more than 68%, whereas the sperm cryopreservation recovery ratio in the LCR group was less than 30% (Figure 2a, Supplementary Table S2).

### 3.2. Proteomic Profile of Spermatozoa

**Figure 3:**
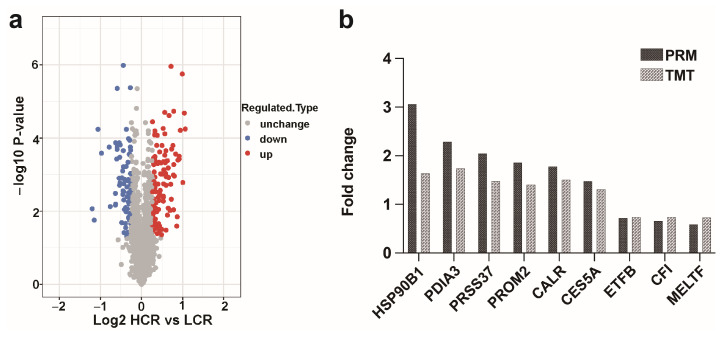
Screening and validation of the DEPs. (**a**) Volcano diagram of DEPs. The horizontal axis is the quantitative value of relative protein after log2 logarithm conversion, and the vertical axis is the *p*-value after log 10 logarithm conversion. A red dot indicates an upregulated protein, and a blue dot indicates a downregulated protein. (**b**) Validation of nine selected proteins in HCR and LCR ram spermatozoa by PRM.

The sperm protein strips of the SDS-PAGE gel were clear and uniform, the protein was not degraded, and there was no significant difference in electrophoretic behavior between the groups (Figure S1); thus, the gel was used for in-gel digestion and peptide extraction. The Pearson’s correlation coefficient of qualified spermatozoa proteins showed that samples from HCR and LCR were highly correlated (Figure 2b). Principal component analysis (PCA) of the quantitative results of the proteins of the three biological replicates of the two groups were separated into two distinct clusters (Figure 2c), which indicated that the samples were suitable for subsequent experiments. A total of 2464 proteins and 18,094 peptides was identified using quantitative TMT-based LC-MS/MS proteomics analysis (Supplementary Figure S2 and Table S3). Between the HCR and LCR groups, a total of 184 differentially expressed proteins were identified with these criteria: at least two peptides, a score > 20, *p* < 0.05, and a fold change of ≥1.2. In total, 112 proteins were more abundant in HCR spermatozoa, while 72 proteins were more abundant in LCR spermatozoa. These identified intergroup DEPs were effectively separated using Rstudio (version 3.5.1, 2018), as shown in the volcano plot (Figure 3a, Supplementary Tables S4 and S5).

### 3.3. Validation of the Selected DEPs by PRM

The PRM assay was used to confirm the authenticity of the results obtained by TMT. The data were presented in Table S6. Since this assay requires the signature peptide of the target protein to be unique, we selected the proteins with a unique signature peptide sequence for the PRM analysis. PRSS37, PROM2, CES5A, PDIA3, HSP90B1 and CALR were selected as upregulated, and MELTF, CFI, and ETFB were selected as downregulated (Figure 3b, Supplementary Table S6). The ratio values showed a trend consistent with the results obtained from the TMT data set, which followed the results of the quantitative analysis of the TMT.

### 3.4. Bioinformatics Analysis of DEPs

**Figure 4:**
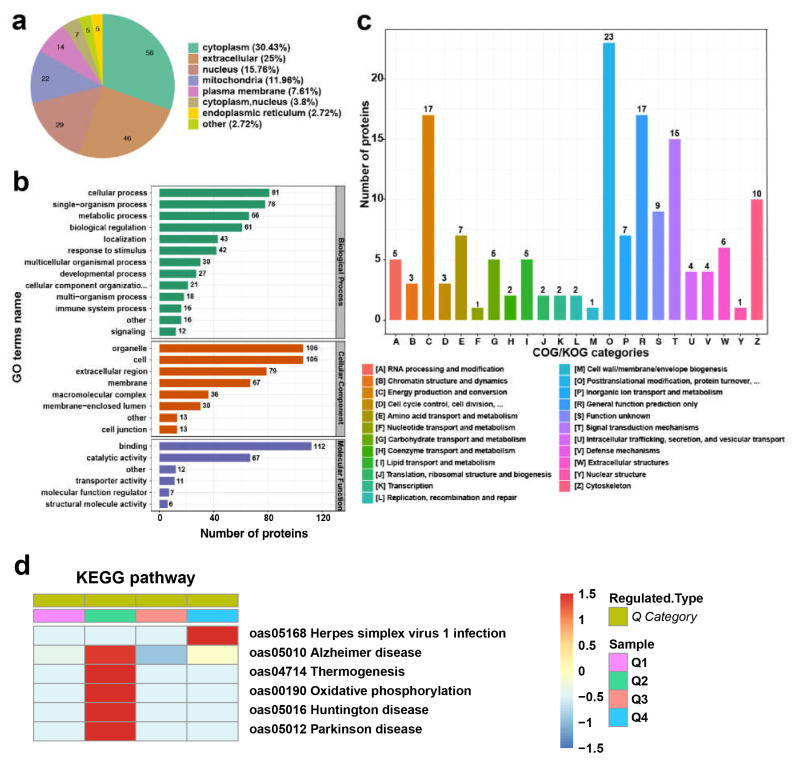
Functional enrichment analysis of different expressed proteins. (**a**) Map of subcellular localization prediction of different expressed proteins. (**b**) GO enrichment analysis of all identified proteins. (**c**) Distribution of COG/KOG functional classification of different expressed proteins. (**d**) Heat map of KEGG pathway enrichment of the downregulated proteins.

We used Wolf PSORT, subcellular localization prediction software, to predict subcellular localization. Figure 4a shows that DEPs were enriched in the following subcellular fractions: cytoplasm (30.43%), extracellular (25%), nucleus (15.76%), mitochondria (11.96%), plasma membrane (7.61%), cytoplasm and nucleus (3.8%), endoplasmic reticulum (2.72%) and other (2.72%) (Supplementary Table S7).

All identified proteins were subjected to a functional analysis based on GO annotation, subcellular localization, and clusters of orthologous groups of proteins (COG). The results of the GO enrichment analysis are displayed in Figure 4b (Supplementary Table S8). The 184 proteins were annotated for biological processes, cellular components, and molecular functions. Results showed that differentially expressed proteins were mainly involved in the response to cellular process (81), single-organism process (78), metabolic process (66), biological regulation (61), localization (43), response to stimulus (42), multicellular organismal process (30), and developmental process (27). The cellular component results showed that the identified proteins were assigned to organelle (106), cell (106), extracellular region (79), membrane (67), macromolecular complex (36), membrane-enclosed lumen (30), cell junction (13), and other (13). Molecular function classification revealed that 6 major processes were discovered from the DEPs, including the binding (112), catalytic activity (67), transporter activity (11), molecular function regulation (7), structural molecule activity (6) and other (12).

The investigation of COG/KOG functional classification analysis identified 23 categories, and the top five categories were post-translational modification, protein turnover, chaperones (23), energy production and conversion (17), general function prediction only (17), signal transduction mechanisms (15), and cytoskeleton (10). Furthermore, related to cellular processes and signaling, metabolism-related functions were enriched, such as cell cycle control/cell division/chromosome partitioning and energy production and conversion (Figure 4c, Supplementary Table S9).

KEGG pathway analysis showed that the downregulated proteins were enriched in five pathways (Figure 4d, Supplementary Table S10), including oxidative phosphorylation (Figure S3) and thermogenesis (Figure S4). Eight proteins (NDUFA8, NDUFB4, NDUFB5, NDUFB7, NDUFS4, ATP5PD, SDHA, and SDHB) were involved in both pathways.

### 3.5. Protein-Protein Interaction Analysis

**Figure 5:**
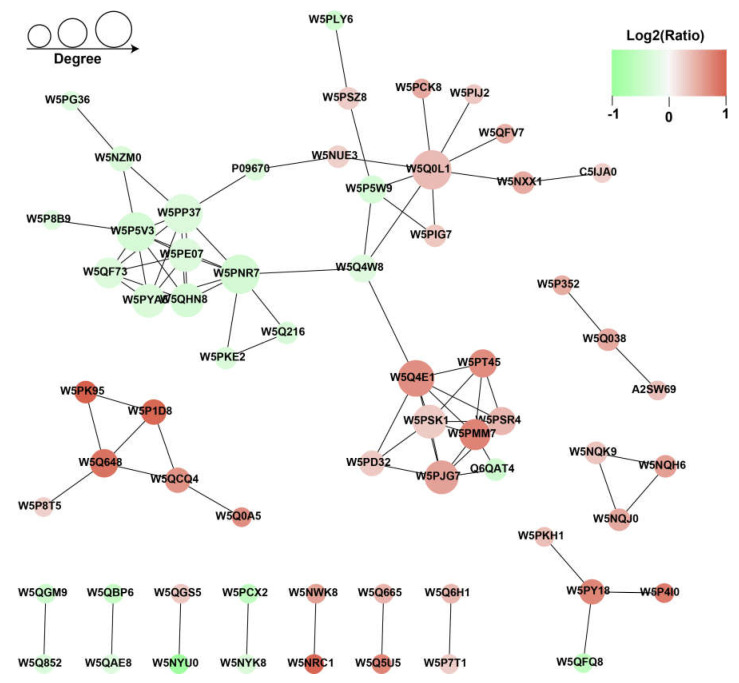
Protein−protein interactions (PPI) network analysis of differentially expressed proteins (DEPs) in HCR vs. LCR comparison. The network was generated using the STRING database.

To better understand the regulation effect of proteins on sperm freezing tolerance, we analyzed the PPI for all DEPs using the STRING software (v.10.5) with a combined score higher than 0.70 (high confidence). We screened out the top 50 proteins with the closest interaction and mapped this to the protein interaction network (Figure 5, Supplementary Table S11). As shown in Figure 5, 63 sperm proteins were connected in this PPI, of which 25 functional proteins were downregulated. NDUFA8, NDUFB4, NDUFB5, NDUFB7, NDUFS4, ATP5PD, SDHA, and SDHB comprised a highly dense protein interaction network and participated in the thermogenesis pathway and oxidative phosphorylation pathway (Figure 4c). The other sub-network was mainly constituted of these proteins: CALR, PDILT, HYOU1, PDIA3, CLGN, MANF, and HSP90B1 (Figure 5).

## 4. Discussion

The freeze–thawing process can damage sperm, and the low cryopreservation recovery ratio of sperm can affect the efficiency of artificial insemination. Sperm cryopreservation recovery ratio is an important indicator for evaluating sperm freezing tolerance and for judging the quality of mammalian sperm. Research increasingly suggests that several proteins function to maintain sperm cryoprotection during the freezing and thawing processes, as well as cell structural integrity and motility [18,19]. Additionally, certain proteins have been investigated as possible indicators for sperm freezability [20]. The main objective of this study was to find new freezing resistance biomarkers by comparing the proteomes of ram sperm with high- and low-cryopreservation recovery ratios. Through TMT-based proteomics methods, we identified 184 DEPs as candidate molecules, and PRM was used to validate the results of the proteomics data.

Bioinformatic analysis indicated key roles of some DEPs in sperm freezing resilience. For example, HYOU1, HSP90B1, PRDX1, and ENO1 were more abundant in the HCR group. HYOU1 and HSP90B1 can all be expressed in the endoplasmic reticulum and play a role in stabilizing and folding other proteins. Hypoxia upregulated protein 1 (HYOU1) was recently demonstrated to be accessible for biotin labeling on the surface of motile human sperm [21], and since inhibition of HYOU1 expression is associated with accelerated apoptosis, it is also suggested that HYOU1 plays an important role in cell protection during hypoxic-induced cell disturbance [22], which suggests that HYOU1 may play an important role in maintaining sperm cell viability. Heat shock protein 90 beta family member 1 (HSP90B1) is a member of the HSP90 family of proteins, most of which are on the endoplasmic reticulum, and it maintains cell activity and immune response [23]. In addition, heat shock protein HSP90 exerts a protective effect in ram sperm cryopreservation, and the sperm cryopreservation recovery ratio could be predicted by detecting the expression of HSP90 [24]. Previous research has shown that telomeres are vulnerable to damage from reactive oxygen species (ROS). Peroxiredoxin 1 (PRDX1) is rich in telomere chromatin, which counteracts ROS-induced telomere damage. This indicates that inadequate PARP1-dependent DNA repair, competition between PARP1 and HR, and ROS-induced telomere catastrophe can occur in the absence of PRDX1 [25]. Therefore, PRDX1 mainly plays an antioxidant protective role in the body. It can also stimulate downstream oxidative stress pathways and remove oxygen free radicals and ROS to prevent cell death [26,27,28]. Enolase 1 (ENO1) is an important metabolic enzyme in the glycolysis pathway, which nutrifies cells by participating in glycolysis, and Eno1 expression is correlated with sperm motility [29,30,31]. In mammals, the primary process for producing energy for sperm motility is glycolysis [32]. Of note, sperm–egg fusion is the primary function of spermatozoa. Interestingly, the expression levels of sperm–egg fusion-related proteins, including CRISP1, CRISP3 and PDIA3, were significantly different in the HCR and LCR comparison. CRISP1 attaches to the heads of sperm and performs its function during sperm–egg fusion [33]. CRISP3 is mainly distributed in the prostate and other tissues and may play a role in sperm maturation [34]. CRISP2 is an important protein that constitutes the dense fiber outside the acrosome and flagellum of spermatozoa and mainly regulates the executive function of the acrosome and flagellum [35]. PDIA3 can catalyze the redox reaction during sperm–egg fusion and participates in the synthesis of glycoprotein by binding with calcium binding protein [36,37]. Consequently, the function of sperm–egg fusion may be an important marker of high sperm cryopreservation recovery ratio; however, the associated functionality is subject to further validation.

Moreover, the PPI network showed that the S100 protein families S100A8 (W5NQK9), S100A9 (W5NQH6), S100A12 (W5NQJ0), S100A14 (W5NWK8) and S100A16 (W5NRC1) presented as functional modules and were more abundant in the HCR sperm. Previous work has revealed that the members of the S100 protein family are involved in calcium ion binding, growth factors, cell viability, signal transduction, transcription, cell survival, and apoptosis, and S100A is mainly responsible for improving thermal stability [38,39]. It is well known that Ca^2+^ signal transduction is the main regulator of sperm flagellum pulsation [40]; it is stored in the sperm neck area for stimulation of flagellum activity in bovine and human sperm [41,42]. A previous study revealed that S100A14 could block Ca^2+^ influx to protect cells from invasion and play a defensive role [43]. Overexpression of S100A16 in lipid cells can promote cell proliferation and differentiation [44]. S100A16 may be a regulator of fat metabolism and has a regulatory effect on glucose and lipid metabolism [45]. Additionally, annexin A2 (ANXA2), which participates in calcium ion binding and signaling receptor binding as well as playing a significant role in the architecture of the cell membrane [46], was found in greater abundance in the HCR group. Increased levels of the A2/S100A10 complex promote cellular functions mediated by the complex, mainly functions pertaining to the cytoskeleton components related to intracellular fusion [47,48]. In our work, S100As and ANXA2 were significantly differentially expressed between HCR and LCR groups, suggesting that a poor cryopreservation recovery ratio in sperm might be caused by a variation of these proteins.

Furthermore, compared with the HCR group, proteins PTGDS, ME3, HSPA9 and VDAC2 were significantly upregulated in the LCR group. PTGDS increases spermatogonia apoptosis in cryptorchid testes of mice [49]. Malic acid enzyme 3 (ME3) mainly assists cell metabolism. NAD(P)+ as a coenzyme of ME3 produces NAD(P)H to generate ROS in mitochondria, which affects sperm motility. HSPA9 has been reported to be a lethal protein that negatively affects cells [50]. The VDAC widely exists in mammalian cell membranes, and mainly comprises three subtypes including VDAC1, VDAC2 and VDAC3, which can support the transmembrane transport of ADP/ATP and NADH [51,52,53]. VDAC2 was enriched in the LCR group, which affected the activity of sperm cells, mainly through the production of ATP in sperm flagella [34]. Ca^2+^ mainly plays a key role in sperm hyperactivity and capacitation, but VDAC2 can regulate Ca^2+^ entry into mitochondria and has similar functions in the sperm flagella membrane and acrosome membrane. We speculated that spermatozoon overactivation could eventually lead to cell death, as Ca^2+^ and ATP can easily cross the membrane due to the presence of VDAC2.

Of note, the mitochondrion is a vital organelle in eukaryotic cells, where it participates in energy metabolism and ATP synthesis [54]. Oxidative phosphorylation is a process that generates ATP through the electron transfer chain (ETC) and is important for maintaining metabolic processes [55]. Our work demonstrated that the oxidative phosphorylation and thermogenesis pathways were enriched in the LCR group. It was found that the dysfunction of mitochondria affects germ cells. As previously reported, both mitochondrial malfunction and disruptions in energy metabolism will promote oocyte aging [56,57]. In particular, the complex proteins involved in the respiratory chain were enriched in the LCR group, which might be the key factor leading to the dysfunction of sperm mitochondria. Nicotine-amide adenine dinucleotide (NADH) dehydrogenase provides electrons in the ETC, and complex I is the largest protein complex in the mitochondrial respiratory chain [58,59]. Complex I (NDUFB4, NDUFB5, NDUFB7), Complex II (SDHA, SDHB) and Complex V (ATP5PD) were enriched in the oxidative phosphorylation pathway, which promotes production of ATP in the mitochondrial oxidative respiratory chain of sperm, resulting in hyperactivation of spermatogenesis. The enrichment of the thermogenesis pathway explains the large-scale production of ATP. Furthermore, Superoxide Dismutase 1 (SOD1) was more abundant in semen with a low cryopreservation recovery ratio, which may be related to the early mitochondrial dysfunction. Enrichment of SOD1 increases the production of hydrogen peroxide, which leads to an increase in cell damage [60]. Furthermore, under normal physiological conditions, the mitochondrial respiratory chain is the primary producer of ROS, and there is a dynamic balance between ROS production and clearance in the cell [61]. In the present study, NDUFB4, NDUFB5, NDUFB7, and SDHB were enriched in the semen of the LCR group, and these proteins participate in the oxidative phosphorylation process to produce ROS. ROS-induced oxidative stress is the primary cause of sperm failure and decreased sperm motility [62].

In summary, several biomarkers that positively affect sperm freezing tolerance were found, which could be exploited as molecular markers for sperm freezing tolerance at the gene or protein level, assisting in the preferred selection of sperm with freezing tolerance preferential for cryopreservation. Furthermore, these biomarkers of sperm freezing tolerance can be introduced to sperm freezing protection solutions as a population or complex, which may improve the freezing recovery of LCR semen.

## 5. Conclusions

Our work provided a TMT-based proteomic technique to identify the DEPs that may affect sperm cryopreservation recovery ratio in rams. The DEPs in spermatozoa were the key factors in regulating a high versus low CR. Interrogation of the protein profile showed that ram HCR spermatozoa were enriched in proteins involved in effectively controlling Ca^2+^ and maintaining flagella structure (S100A8, S100A9, S100A12, S100A14 and S100A16), antioxidant protection and anti-apoptosis (HYOU1, PRDX1) to prevent cell death, and maintaining cell activity and immune response (HSP90B1). We also revealed that the dysfunction of the mitochondrial respiratory chain might affect the cryopreservation recovery ratio of sperm (NDUFB4, NDUFB5, NDUFB7, and SDHB), which may be a biomarker of LCR spermatozoa in rams.

## Figures and Tables

**Table 1:** Sperm concentration and body condition characteristics of HCR and LCR rams.

Group	Concentration (Million/mL)	Age (Months)	Weight (kg)	Breed
HCR	3861.60 ± 296.11	37.00 ± 0.58	98.33 ± 0.88	Dorper sheep
LCR	4191.53 ± 314.09	36.33 ± 0.88	96.33 ± 0.88	Dorper sheep

**Table 2:** Differences in the quality parameters of semen in HCR and LCR. Values are presented as mean ± SEM.

Parameters	HCR	LCR	*p*-Value
Fresh sperm			
MOT (%)	82.3 ± 0.006	80.9 ± 0.02	0.41
VCL (μm/s)	101.3 ± 2.98	103.1 ± 3.10	0.70
VSL (μm/s)	43 ± 2.65	38.725 ± 2.14	0.25
VAP (μm/s)	60.8 ± 1.33	58.1 ± 1.43	0.18
LIN (%)	42.8 ± 2.71	37.9 ± 2.34	0.21
STR (%)	63.8 ± 2.17	60.6 ± 1.84	0.31
ALH (μm)	3.6 ± 0.16	3.8 ± 0.18	0.33
BCF (Hz)	6.2 ± 0.15	6.1 ± 0.20	0.60
Cryo-recovered sperm			
MOT (%)	56.6 ± 0.02 ***	22.1 ± 0.02 ***	<0.001
VCL (μm/s)	80.2 ± 1.94 ***	48.5 ± 2.49 ***	<0.001
VSL (μm/s)	31.5 ± 1.27 ***	22.2 ± 1.33 ***	<0.001
VAP (μm/s)	45.4 ± 1.50 ***	36.9 ± 2.06 ***	<0.001
LIN (%)	39.7 ± 1.95 *	45.7 ± 1.72 *	<0.05
STR (%)	61.8 ± 1.38 *	67.1 ± 1.42 *	<0.05
ALH (μm)	3.3 ± 0.12 ***	2.6 ± 0.09 ***	<0.001
BCF (Hz)	5.1 ± 0.11 ***	6.9 ± 0.25 ***	<0.001

Note: The data expressed were the means of four experimental replicates with three samples per replicate. Asterisks denote values that were different between the spermatozoa of HCR and LCR (* *p* < 0.05, *** *p* < 0.001 following one-way ANOVA with Duncan’s post hoc analysis). MOT = motility (%); VCL = curvilinear velocity (µm/s), VSL = straight-line velocity (µm/s), VAP = average path velocity (µm/s), LIN = linearity (%), STR = straightness (%), ALH = mean amplitude of head lateral displacement (µm), BCF = beat cross frequency (Hz).

## Data Availability

All data generated and analyzed during this study are included in this published article. Raw data supporting the findings of this study are available from the corresponding author on request.

## Supplementary Materials

The following supporting information can be downloaded at: https://www.mdpi.com/article/10.3390/ani13142368/s1, Figure S1: Map of SDS-PAGE; Figure S2: Basic statistical chart of mass spectrometry data; Figure S3: Oxidative phosphorylation pathway; Figure S4: Thermogenesis pathway; Table S1: Sperm concentration and body condition characteristics of HCR and LCR rams. Table S2: Sperm motility parameters; Table S3: Data set of all identified proteins in two group samples (HCR vs. LCR); Table S4: Category details of 112 proteins of HCR spermatozoa; Table S5: Category details of 72 proteins of LCR spermatozoa; Table S6: Validation data of selected DEPs by PRM; Table S7: DEPs subcellular localization information; Table S8: GO annotation information of DEPs; Table S9: Category classification information of COG terms of DEPs; Table S10: Enrich downregulated protein information of KEGG pathway; Table S11: Protein information involved in PPI network.
