# Supplementary figures and images for: Identification of Biomarkers Affecting Cryopreservation Recovery Ratio in Ram Spermatozoa Using Tandem Mass Tags (TMT)-Based Quantitative Proteomics Approach
Source: Animals (Basel). 2023 Jul 20;13(14):2368. doi: 10.3390/ani13142368 (PMC10376853; doi:10.3390/ani13142368)

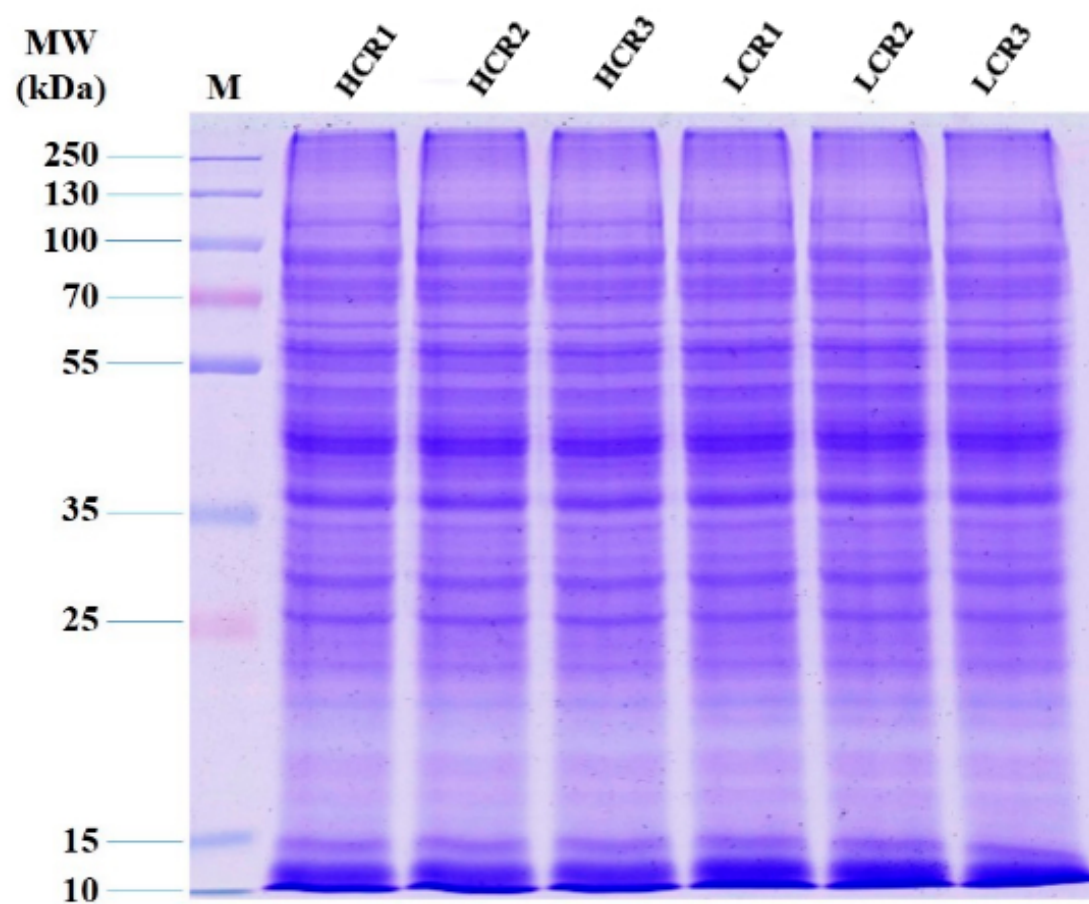

Fig. S1. Map of SDS-PAGE

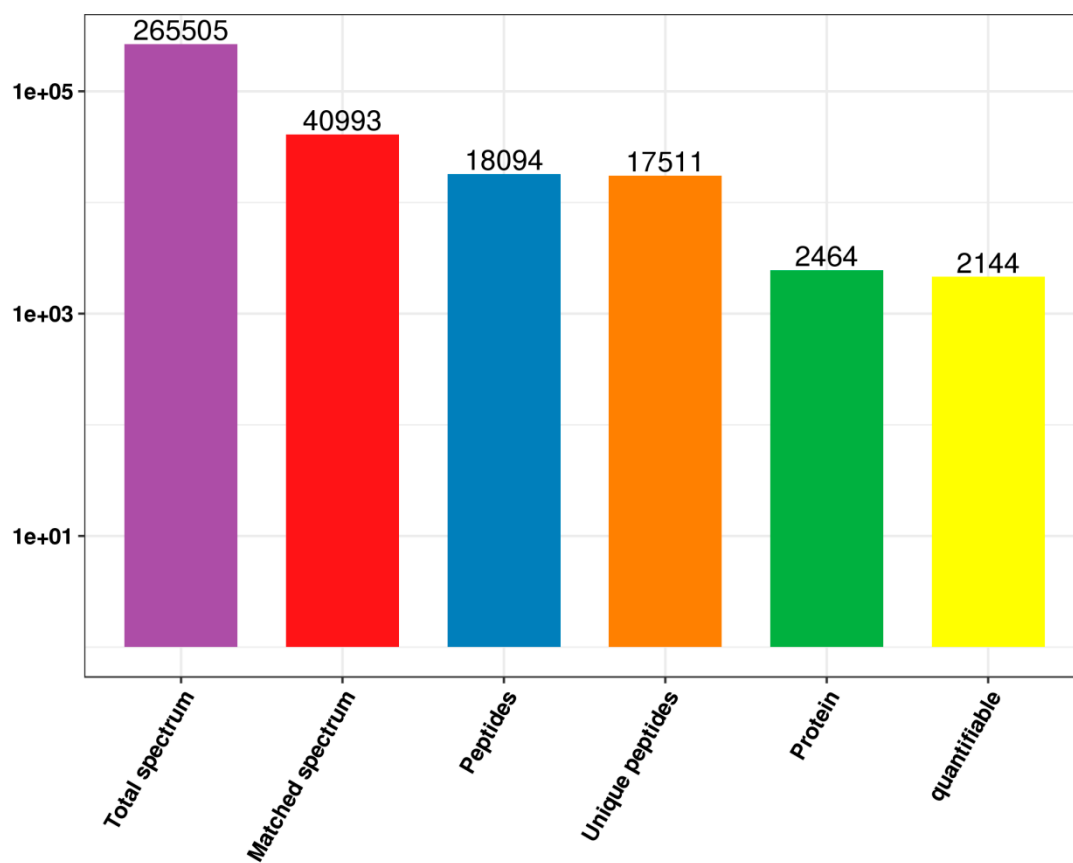

**Fig. S2. Basic statistical chart of mass spectrometry data.**

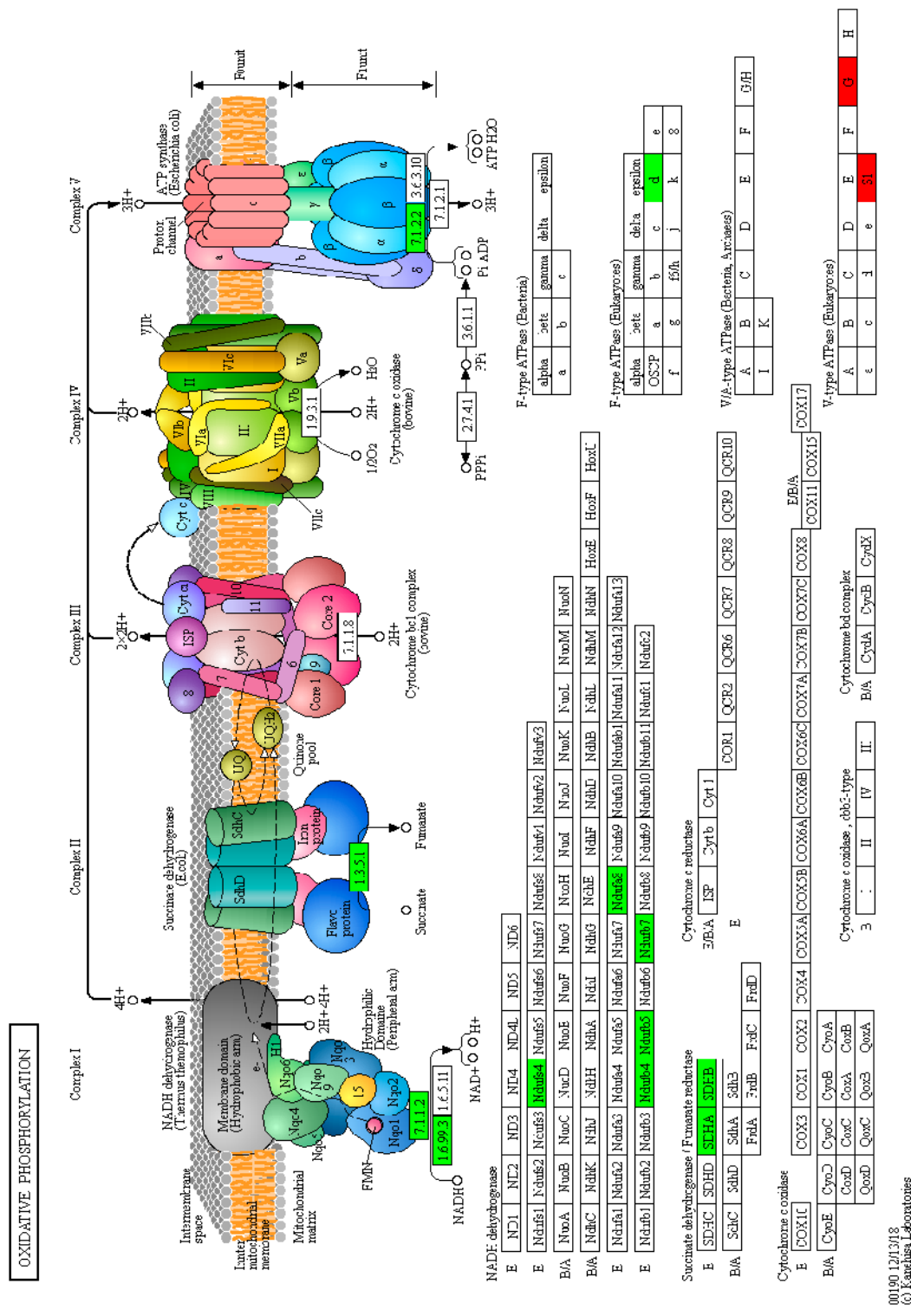

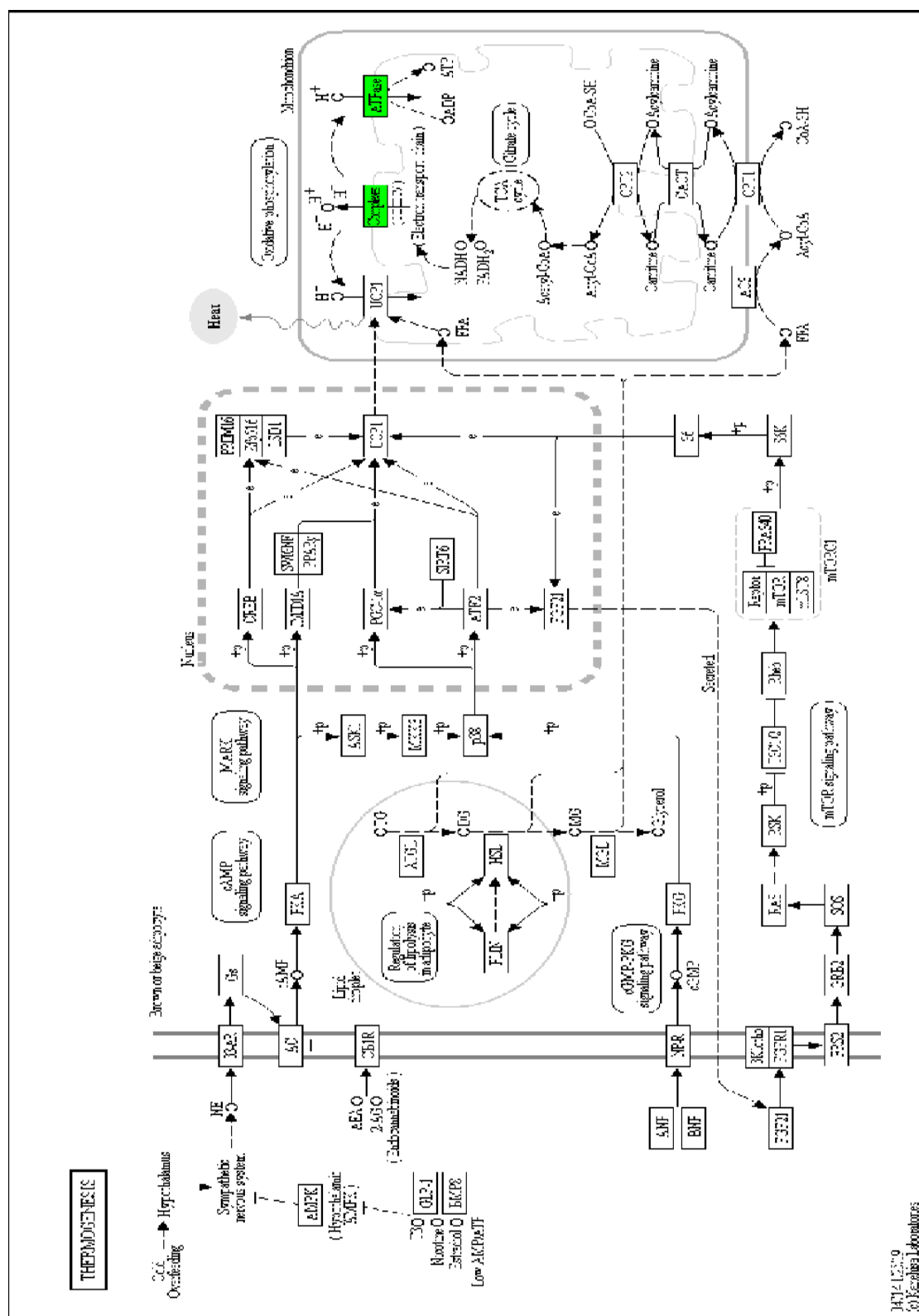

Supplement: Supplementary file 1 [file animals-13-02368-s001.zip › Figures S1-S4.pdf]
